# Space Breeding: The Next-Generation Crops

**DOI:** 10.3389/fpls.2021.771985

**Published:** 2021-10-27

**Authors:** Tapan Kumar Mohanta, Awdhesh Kumar Mishra, Yugal Kishore Mohanta, Ahmed Al-Harrasi

**Affiliations:** ^1^Natural and Medical Sciences Research Center, University of Nizwa, Nizwa, Oman; ^2^Department of Biotechnology, Yeungnam University, Gyeongsan, South Korea; ^3^Department of Applied Biology, School of Biological Science, University of Science and Technology, Ri-Bhoi, India

**Keywords:** space, breeding, crop, microgravity, cosmic radiation

## Abstract

Since the beginning of space exploration, researchers have been exploring the role of microgravity, cosmic radiation, and other aspects of the space environment on plant growth and development. To create superior crop varieties and achieve noticeable success in the space environment, several types of research have been conducted thus far. Space-grown plants have been exposed to cosmic radiation and microgravity, which has led to the generation of crop varieties with diverse genotypes and phenotypes arising from different cellular, subcellular, genomic, chromosomal, and biochemical changes. DNA damage and chromosomal aberrations due to cosmic radiation are the major factors responsible for genetic polymorphism and the generation of crops with modified genetic combinations. These changes can be used to produce next-generation crop varieties capable of surviving diverse environmental conditions. This review aims to elucidate the detailed molecular mechanisms and genetic mutations found in plants used in recent space crop projects and how these can be applied in space breeding programmes in the future.

## Introduction

**Figure 1:**
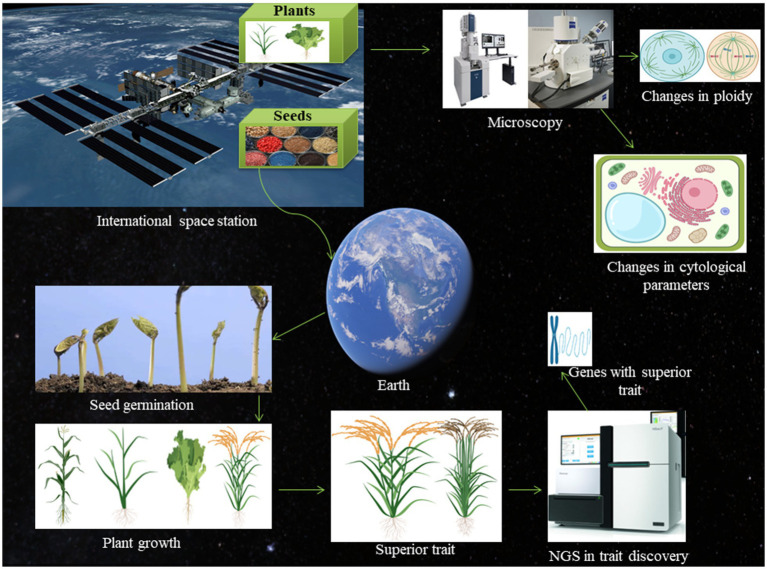
Schematic presentation of the space-breeding platform. The international space station is well-known for its role in space biology research. The plant seeds can be exposed to microgravity and cosmic radiation in space and brought back to the earth and sow for germination. The phenotypic and genotypic parameters can be measured to find the superior trait. The superior trait can be subjected to next-generation sequencing to find potential gene associated with the space environment. Once the gene will be identified, it can be used for molecular breeding to generate crops with the superior trait. The seeds can also be germinated in the space environment itself and grown plants can be brought back to the earth to check for chromosomal aberration, changes in cell shape and size, and alteration in the cell division.

Currently, space research has gained widespread attention and there have been considerable advances in in-depth space exploration (Frisbee, 2003; Anqi, 2021; Young and Sutton, 2021). Beyond the ozone layer, space gradually loses gravity, magnetic field, air, and environmental pressure (Eichinger et al., 2020). As a result, it receives large quantities of cosmic radiation, the effects of which are still not well understood (Bagshaw, 2008; Zhang et al., 2014). Additionally, space is a hyper-vacuum environment. Some scientists are working towards establishing life on Mars, and extensive laboratory and other researches have been conducted in order to do so (McKay, 1998, 2010; Simoneit et al., 1998; Westall et al., 2013). However, the absence of a magnetic field and other factors on Mars makes it difficult for plants to grow there (Acuña et al., 2001; Connerney et al., 2001). The International Space Station (ISS) an artificial habitable satellite that was launched and established in 1998 and is currently functioning very well (Thirsk et al., 2009). It has been used as a research centre for astrobiology studies in a microgravity environment (Figure 1; Penley et al., 2002; Jules et al., 2004; Rabbow et al., 2009, 2012, 2015). Space travel is not very quiet, as the noise generated by the spacecraft remains constantly high. Moreover, the higher sound frequency can be a deterrent to living cells. It was recently reported that China harvested the first batch of rice that it is calling “space rice” or “rice from heaven” (Anqi, 2021). During the 23days of China’s Chang’e-5 mission to lunar orbit, they had sent 40g of rice with it (Ghosh, 2021). Upon returning to Earth and completing the lunar mission, the rice grains were grown in the lab, and then in the field. Seeds were harvested at the South China Agriculture University, Guangzhou. The seeds were 1cm long and the best seeds were identified through genomic selection (Voss-Fels et al., 2019) and bred in laboratories for further investigation. During the journey in space, the rice seeds were exposed to cosmic radiation and may have undergone intense mutation that could potentially lead to higher yields. The space environment may influence plant growth and development and cause changes in the genetic makeup of the seeds, which will likely experience an increase in chromosomal aberrations when they are transported into space, and this could be very useful in terms of crop breeding (Resende et al., 2021). We discuss here some details about the phenotypic, genomic, and molecular events that plants incur on exposure to the space environment.

## Cell Size and Differentiation

The structure of a cell may change upon exposure to the external environment of space. For example, round cells were found in the cotyledon of pine, as well as in the root meristem of *Crepis* and maize (Il’ne and Parfenov, 1979). In microgravity, there is increased surface tension, which may result in rounded cells and reduced mechanical pressure on the attachment of cells (Il’ne and Parfenov, 1979). The moss *Funaria hygrometrica* grown in space for 96days contained more pear-shaped and dumbbell-shaped cells than the control moss grown on Earth. Furthermore, smaller cells, reduced volumes of starch granules, and altered chloroplast structures have been observed (Kordyum et al., 1981). Cells were found to be larger in the roots of *Crepis*, maize, pea, and wheat (Saunders, 1971; Merkys et al., 1975, 1976). An increase in cell elongation has been observed in wheat and maize seedlings (Merkys et al., 1976; Il’ne and Parfenov, 1979). On shuttle mission D-1, anise seeds were grown for somatic embryogenesis, and it was found that the somatic embryo cells differentiated more rapidly in space. Within 4days of growth in the space-grown culture, 90% of the cells displayed polarity and leaf or root primordia, whereas the control showed polarity after 6days. A larger differentiation zone was observed in carrot somatic embryos, resulting in longer roots (Il’ne and Parfenov, 1979). The space-grown plants received more sunlight than the ground-grown plants, which might explain why they generated chlorophyll earlier than ground-grown plants (Theimer et al., 1986). As the cells did not undergo normal elongation, they underwent early differentiation, leading to the formation of early root hairs near the tip of the roots (Merkys et al., 1983). Oat seedlings grown in the SL-2 spacecraft showed an increased number of lateral roots, which might be due to a reduced rate of cell division in the root tips (Halstead and Dutcher, 1987). The internode of pea seedlings grown in space was found to be extremely elongated compared to that of the control. The growth and development of orchid plants in space have been reported to be retarded. After the initiation of the space journey, the flowers suffered severe damage, cessation of growth, and death (Halstead and Dutcher, 1987). Despite the roots, stems, and leaves of orchid plants continuing to grow and develop, their flowers did not appear even after 6months (Gorkin et al., 1980). *Arabidopsis* plants grew very slowly, taking 28days to achieve the rosette stage and 44days to initiate flowering, while in the control plant they took 13 and 36days, respectively (Merkys and Laurinavichius, 1983).

## Cell Division

For many years, induced mutations have been one of the most successful breeding strategies for plant breeders. Currently, there are a large number of mutant crop varieties worldwide, and their numbers are continuing to rise. Nevertheless, the space breeding approach of mutation through cosmic radiation is a recent development (Anqi, 2021). The space environment has microgravity, a hyper-vacuum, a weak geomagnetic field, and absence of microbes, which makes it an ideal environment for breeding plants (Paul et al., 2013; Kiss, 2014; Kordyum, 2014). Seed germination and plant growth have been found to be affected by the space environment in previous studies (Dutcher et al., 1994; Liu and Zheng, 1997). Compared to the control on Earth, mitotic cell division decreased with an increasing frequency of chromosomal aberrations. This spectrum of mutagenic effects has been reported to be universal among plants because of the mutagenic effects of space conditions (Liu et al., 2009). A reduction in cell division has been reported in plants grown in Biosatellite II (Thimann, 1968; Dubinin et al., 1977). Seedlings of *Pisum sativum* and *Crepis capillaris* grown during Soviet flights exhibit reduced cell division (Merkys et al., 1976; Tairbekov et al., 1979; Halstead and Dutcher, 1987). Oat seedlings grown on shuttle missions SL-2 and STS-3, and *Helianthus annuus* plants grown on STS-2 and STS-3 had 10% fewer dividing cells than the ground-control plants (Merkys et al., 1983; Sytnik et al., 1983). Reduced mitotic cell division of sunflowers, mung beans, and oats was found at the anaphase and telophase. A longer duration of space flight might have led to a greater reduction in cell division, in which the maximum number of cells was arrested at the metaphase of the cell cycle (Halstead and Dutcher, 1987). However, in lettuce, when cell division was reduced most cells were at the telophase (Merkys et al., 1983). The presence of multiple nuclei in *Crepis* and *Pisum* seedlings has been reported for flights conducted by the Soviet Union, while a sample of sunflowers grown on STS-2 revealed aneuploidy (2n−1=33) in root cells (Krikorian and O’Connor, 1984). Mitotic disturbances, including abnormal migration of the nucleus, were also found in the microspores of *Tradescantia paludosa* (Delone et al., 1964, 1966). Similarly, chromosomes were bunched at metaphase, as opposed to being located in the equatorial plate. A change in the spindle axis direction, unseparated chromatids, 3- and 4-pole mitoses, and non-disjunction of chromosomes were observed (Delone et al., 1965). It is possible that such incorrectly separated chromatids can produce the aneuploid genome that was observed in sunflowers. Fused embryo sacs have been identified in *Tradescantia* plants with clumped and scattered nuclei (Saunders, 1971). This could be caused by a malfunction in spindle organisation. Additionally, the root tips showed abnormal spindle organisation, multinucleated cells, and abnormal nuclei (Saunders, 1971).

## Subcellular Changes

Weightlessness and microgravity play crucial roles in the subcellular organelles that make up a cell (Morrison, 1994; Bradbury et al., 2020). The cell walls of higher plants grown in a weightless condition were approximately one-third as thin as those of control plants (Dubinin, 1984; Laurinavichius et al., 1984). Plants grown in space for 24days had 54% lower cellulose content in their cell walls, while mung bean seedlings had 18% lower lignin content (Halstead and Dutcher, 1987). A reduction in protein and enzyme levels was also observed in space-grown seedlings (Cowles et al., 1984). We have already mentioned a reduction in mitosis cell division and chromosomal aberration in space-bound plants. The roots of *Arabidopsis* and pea plants grown in space had abnormal nuclei distribution and condensed chromatin in the statenchyma cells (Kordyum and Sytnik, 1983). The number of nucleoli was reduced in space-grown pine cells, whereas a significant increase in nucleus volume was observed in wheat seedlings. However, no changes were observed in the root caps of maize (Moore et al., 1987a). The location of the nucleus remained unaffected in cress, lentil, and lettuce, suggesting genetic programming of the nuclear organisation in the cell (Merkys et al., 1983; Perbal et al., 1986; Volkmann et al., 1986). Space-grown cells exhibited random distributions of amyloplasts in the root cap, whereas the 7-day-old seedlings showed a clearer stroma in the amyloplast and decreased starch grains and plastid reticulum. Most of the plastids post-18days contained a centrally located single starch granule, while others contained few or no starch grains (Sytnik et al., 1984). Maize seedlings at 4.8days had less starch in their columella cells than control plants. In a leaf sample of space-grown pea plants at 29days, disintegration of grana, separation of intergrana, shrinkage of the stack of grana, and formation of electron transport vesicles were observed (Halstead and Dutcher, 1987).

The electron density of the endoplasmic reticulum (ER) cisternae was reduced in the columella cells of space-grown *Arabidopsis*, cucumber, and pea seedlings (Halstead and Dutcher, 1987). In other cases, rounding and reduction of cisternae have been reported, whereas the disappearance of rough ER and ribosomes has been reported in *Funaria* protonema (Sytnik et al., 1984). While the maize seedlings germinated on Earth and grown in space had normal ER distribution in columella cells, those grown in space had spherical and ellipsoidal ER clustered at their periphery (Moore et al., 1987b). This reflects the effect of gravity on the distribution and integrity of the ER. The root caps of *Arabidopsis*, maize, pea, and seedlings with less-ordered membranes showed swollen mitochondria with electron-dense matrices (Il’ne and Parfenov, 1979; Kordyum and Sytnik, 1983). In the pea root meristem, dictyosomal cisternae underwent morphological changes as a result of weightlessness or microgravity, and there was a 50–90% reduction in dictyosome volume in the calyptrogen, columella, and peripheral cells of the root cap compared to the control (Moore et al., 1987a). In addition, mucilage secretion was considerably reduced in pea and maize seedlings (Tairbekov et al., 1986; Moore et al., 1987a).

## Genetics and Polymorphism

Plant cultivated in space, the space environment can undergo changes during their development. In one experiment, *Arabidopsis* plants were carried into space at the cotyledon stage, and these plants succeeded in growth and flowering. However, the androecium and gynoecium of these plants were degenerated and sterile. Although the gross morphology of the reproductive organs was not affected, their sterility was caused by unsuitable illumination (Kordyum and Chernyaeva, 1982; Kordyum et al., 1983; Dubinin, 1984; Kostina et al., 1984). Afterwards, the plants were planted and sent back to Earth, where they completed flowering; however, their fertility was reduced, the frequency of recessive mutants increased, and germination success was reduced (Kostina et al., 1984, 1986). Eventually, the seeds from these *Arabidopsis* plants were grown in space to complete their life cycle. Nonetheless, the plants appeared shorter than normal, produced fewer leaves, and had smaller and fewer siliques (Halstead and Dutcher, 1987). When the F1 seeds obtained from the flight were planted in the ground, they showed reduced germination potential, with smaller hypocotyl and cotyledonary leaves. However, 42% of the seeds derived from the flight were biologically completed and produced fertile plants. Compared to the control plants, the F2 plants grown on Earth did not show any significant changes in developmental morphology (Parfenov and Abramova, 1981; Halstead and Dutcher, 1987).

The space environment induces mutations in crop genomes. A random amplified polymorphic DNA (RAPD) study of 201 rice germplasms developed from space-flown seeds revealed genomic polymorphisms. An estimated 30.2% genetic polymorphism was recorded from space-environment-induced mutations in rice, when compared to the ground control (Luo et al., 2007). However, the growth and fertility of all plants returned from the spaceships and the ground control plants were functionally normal (Luo et al., 2007). Studying 24 random primers led to 189 loci being detected, of which 4–15 loci were detected per primer. Fragments of the amplicon were between 400 and 2,000 base pairs (Luo et al., 2007). When rice seeds I-B11 DNA were amplified with OPS-19 as a primer, the sequencing report showed the presence of at least 12 single nucleotide polymorphisms (SNPs) in comparison with control samples (Luo et al., 2007). SNPs were also observed in OPN-9-B16 and OPB-13-A4 varieties (Luo et al., 2007). For successive generations, genetic polymorphisms were also examined for rice seeds I-B11 and I-B15. In total, 121 polymorphic loci were identified by polymorphism analysis using 29 random primers (Luo et al., 2007). Analysis revealed that polymorphism from individual plants and their progenies varies greatly compared to ground-based control (Luo et al., 2007). For I-B11, nine polymorphic loci were found in the first, whereas nine polymorphic loci were detected in third, fourth, and fifth generation (Luo et al., 2007). The field study revealed that plants with space-bound seeds grew shorter than their wild-type counterparts, QHZ. However, the progenies of I-B11 from the second generation headed 8–10days earlier than the control QHZ.

*Oryza sativa* lines R998, Gui99, and Hanghui No. 7 (restorer lines) were subjected to space mutation, and 104 pairs of SSR primers were used to check the polymorphisms; this revealed the presence of at least 2,538 polymorphic loci (Wu et al., 2011). The frequency of polymorphisms was 9.85%, which was caused by small fragments of DNA deletion or insertion mutations (Wu et al., 2011). The restoring and combining abilities of the mutants were evaluated through crossing with diverse sterile lines. The results showed that the space mutagenic effect has the potential to cause variations in the restoring ability of restorer lines in perpetuity (Wu et al., 2011). The pollen sterility in F_1_ hybrid combinations showed considerable variation compared to the control and some restorer lines mutated into the maintainer lines (Wu et al., 2011). The GCA values of Hanghui No. 7-4, Gui 99-4, Gui 99-2, R998-3, and R998-4 were higher. Genetic polymorphisms in maize male sterile mutants were also analysed following space exposure (Zhang et al., 2012). Analysis of amplified fragment length polymorphism using 56 primer pairs led to amplification of 32 primer combinations. Sequencing of the eluted fragments led to identification similarity with the expressed sequence tag gi33771201 of the maize sperm library (Zhang et al., 2012). The DNA sequence AC187265 was found to be located on the third chromosome (Zhang et al., 2012).

In space-grown lucerne seedlings, peroxidase activity was increased by 18% compared to the ground control (Li et al., 2004). This might be due to the activation and accumulation of oxygen species induced by space flight factors. The gel electrophoresis band patterns of the peroxidase were also changed in the space-grown seedlings, resulting in a few new bands (Li et al., 2004).

## Chromosomal Aberrations

The radiation encountered in space leads to various types of DNA damage in cells: single nucleotide damage, single-stranded breaks, and double-stranded breaks (Furukawa et al., 2020). Double-stranded DNA breakage is one of the most common and severe forms of DNA damage. This leads to the activation of several DNA damage repair pathways about genomic stability. If a double-stranded break is not repaired properly, it may lead to cellular senescence and cell death (Furukawa et al., 2020). Usually, space-mediated DNA damage is not repaired properly because of the heavy energy radiation of α-rays and others, leading to failure in the mechanism of non-homologous end joining for DNA damage repair (Mohanta et al., 2017a). In such a situation, the homologous recombination mechanism is activated, leading to damage repair. As previously discussed, space-grown plants often develop chromosomal aberrations, which can be stable or unstable. The unstable chromosomal aberrations are categorised as ring, rearranged acentric, and multicentric chromosomes (Furukawa et al., 2020) and always remain unrepaired. Unstable chromosomal aberrations are usually lost during cell division due to their impaired DNA replication fork, broken termini without telomeres, and loss of chromosomal segregation mechanisms. The dicentric chromosome, which is the most common type of unstable chromosomal aberration, can be easily identified because of the presence of two centromeres (MacKinnon and Campbell, 2011; Stimpson et al., 2012; Furukawa et al., 2020). Stable chromosomal aberrations contain monocentric chromosomes that can be transmitted to daughter cells and hence can be used as a biomarker of past exposure to cosmic radiation.

Chromosomal aberrations have been found in most cases in seeds that have been taken into space and subsequently germinated (Delone and Antipv, 1967; Delone et al., 1971). Root meristematic cells were more frequently found to exhibit chromosomal aberrations. In *C. capillaris*, the amount of chromosomal aberration was increased, and in *Arabidopsis thaliana*, the viability and germination of seeds were significantly decreased. When grown in space, *Arabidopsis* plants showed abnormal growth in their reproductive systems and seeds; the germination potential, survival, and fertility were also reduced (Halstead and Dutcher, 1987). All of these events were attributed to chromosomal aberrations in meristematic stem cells. As metabolising cells, these are more prone to cosmic radiation exposure in space flight than dormant seeds. After exposure to space radiation for 3–234days and germination in space, *Crepis* seeds showed more aberrations than their ground-germinated counterparts (Kostina et al., 1984). Seedlings of *Pinus sylvestris*, however, did not show such an increase in chromosomal aberrations (Il’in and Parfenov, 1979; Tairbekov et al., 1979). The main reason for chromosomal aberrations, however, is extremely unlikely to occur in a short flight where cosmic radiation is quite low (Aliyev et al., 1985). Radioprotectors, such as 5-methoxytryptamine, aminoethylisothiourea, and cysteine, have not been able to reduce space-induced chromosomal aberrations in barley (Nuzhdin and Dozotseva, 1972; Nuzdhin et al., 1975). An 827-day exposure resulted in a significant increase in chromosomal aberrations in *Crepis* seeds, although there was virtually no effect on *Arabidopsis* seed germination potential (Anikeva et al., 1983; Halstead and Dutcher, 1987).

## Elemental Composition

The exposure of plants to space has resulted in several physiological, cellular, genomic, and molecular changes. As a result, the micro-and macro-elemental composition of the cell may change. For example, pea plants grown on the Salyut space station showed problems with mineral balance, resulting in an increase in phosphorus and potassium levels in the shoots, but a sharp decrease in calcium, iron, magnesium, manganese, and zinc levels (Dubinin, 1984). It is most likely that a reduction in elemental composition is related to a reduction in root growth. In calcium signalling events, calcium ions act as a second messenger, and its reduction in the cell explains the lower metabolic, cellular, and physiological events in the cell (Mohanta et al., 2012, 2015, 2017b, 2019; Mohanta and Sinha, 2016). The presence of manganese during photosynthesis contributes to the hydrolysis reaction, and reduced manganese in cells is directly related to a reduction in photosynthetic activity. Furthermore, in an analysis of 5-day-old pea seedlings, ATPase activity was inhibited by reduced Ca^2+^, Mg^2+^, and K^+^ levels, which suggests that this is a further effect of microgravity. The inhibition of ATPases leads to reduced protein content in plasmodesmata. It is most likely that the decreased ATPase activity was due to a considerable reduction in Ca^2+^-dependent ATPases. In the control group, Ca^2+^ was found in the plasmodesmata and plasmalemma, whereas in the treated group, Ca^2+^ disappeared from these organelles but was found in the plastid, ER, dictyosome, and nuclear membrane (Palladina et al., 1984). From these reports, it can be speculated that free cellular calcium is more likely to be membrane-bound under hypogravity. Changes in Ca^2+^ levels also trigger the activation of membrane phospholipases (Palladina et al., 1984). Consequently, active and passive Ca^2+^ transport is disrupted because of reduced Ca^2+^ ATPase activity.

## Development of Mutant Varieties Through Space Breeding

Plants grown under zero gravity and cosmic radiation undergo physical, physiological, and genetic changes (Krikorian and Steward, 1978; Böhmer and Schleiff, 2019), and it is possible that cosmic radiation may be used as a method of genetic modification. Space crop projects have garnered enormous attention worldwide for a long time and have succeeded in revealing a considerable amount of biological information (Ferl et al., 2002; Wolverton and Kiss, 2009). It has been reported that China has been quite active in such a mission since 1987, and as of now, has developed approximately 200 plant varieties caused by space radiation (Zhihao, 2020). High-altitude balloons are also used by China to conduct breeding experiments in space. The launch of Shiijian-8, the first space breeding satellite, took place on 9 September 2006 (Chen, 2019). It contained approximately 2,000 plant accessions from 133 plant species. Seed germination of cotton, maize, sunflower, cucumber, tomato, wheat, barley, and soybean noticeably increased after space flight, but rice, millet, pea, sweet pepper, tobacco, and lettuce failed to show any differences (Liu et al., 2009). The seed germination potential of eggplant, radish, watermelon, and sorghum was reduced, whereas that of wheat, barley, and triticale was significantly higher than that of ground control and gamma-irradiated seeds (Liu et al., 2009). The activities of both esterase and peroxidase enzymes in these species were also increased during flight (Liu et al., 2009).

At least 66 mutant crop varieties, including lucerne, cotton, pepper, sesame, rapeseed, rice, tomato, and wheat, have been released in China through space breeding programmes (Liu et al., 2009). Although developing plant varieties that thrive in microgravity and resist cosmic radiation may be an important goal for the scientific community, an undesirable mutation in the genome could have deleterious effects on other crop varieties. It is necessary to consider the possibility of cross-contamination of deleterious traits between varieties. Therefore, the conduct of such research should be subject to strict international regulations to avoid the possibility of unexpected results.

## Conclusion and Future Perspectives

Despite advances in conventional and molecular breeding in agricultural research, genetically modified crops are not widely accepted. Therefore, it remains uncertain how society will react to crops that have been genetically modified in space, specifically, the “space rice” released by China. Genome sequencing and transcriptome sequencing should be performed to better understand the genomic variations caused by cosmic radiation. It is important to perform further investigations to understand how the proteome and metabolome contents will be affected. In addition, SNPs and genome-wide association studies can aid in identifying potential genetic markers associated with space breeding programmes. Once a particular gene/locus is identified, its role can be expanded to other crop varieties. This information can be used in simulation models for further application in other crop varieties using mutations and chromosomal aberrations that occur in different crop species. Statistical software such as EnvRtype can be useful for analysing large-scale environmental data for quantitative genomics and environment-based ecophysiological interactions (Costa-Neto et al., 2021). Interconnecting genomic, high-throughput phenotyping, and environmental parameters (Crossa et al., 2021) can be very useful in addressing space breeding strategies. Modelling studies can be performed using genomic and space environment data for selection (Jarquín et al., 2014; Marcatti et al., 2017) and to identify better traits for crop production. The single-step genomic and pedigree×environment interaction model can extend the genomic relationship information on individual genotypes to pedigree information from multiple phenotyped species (Pérez-Rodríguez et al., 2017). The genomic relationship of individual species can be combined to obtain the desired traits for breeding programmes.

## Author Contributions

TM conceived the idea, surveyed literature, and drafted and revised the manuscript. AM, YM, and AA-H revised the manuscript. All authors contributed to the article and approved the submitted version.

## Conflict of Interest

The authors declare that the research was conducted in the absence of any commercial or financial relationships that could be construed as a potential conflict of interest.

## Publisher’s Note

All claims expressed in this article are solely those of the authors and do not necessarily represent those of their affiliated organizations, or those of the publisher, the editors and the reviewers. Any product that may be evaluated in this article, or claim that may be made by its manufacturer, is not guaranteed or endorsed by the publisher.
